# Adenovirus platform enhances transduction efficiency of human mesenchymal stem cells: An opportunity for cellular carriers of targeted TRAIL-based TR3 biologics in ovarian cancer

**DOI:** 10.1371/journal.pone.0190125

**Published:** 2017-12-21

**Authors:** Lindsay M. Kuroki, Xingjian Jin, Igor P. Dmitriev, Elena A. Kashentseva, Matthew A. Powell, David G. Mutch, Allan B. Dietz, David T. Curiel, William G. Hawkins, Dirk Spitzer

**Affiliations:** 1 Division of Gynecologic Oncology, Department of Obstetrics and Gynecology, Washington University School of Medicine, St. Louis, MO, United States of America; 2 Alvin J. Siteman Cancer Center, St. Louis, MO, United States of America; 3 Department of Surgery, Washington University School of Medicine, St. Louis, MO, United States of America; 4 Department of Radiation Oncology and Biological Therapeutics Center, Washington University School of Medicine, St. Louis, MO, United States of America; 5 Department of Laboratory Medicine and Pathology, Mayo Clinic, Rochester, MN, United States of America; University of Miami School of Medicine, UNITED STATES

## Abstract

Clinical application of tumor necrosis factor-related apoptosis-inducing ligand (TRAIL)-based cancer therapeutics has not reached optimal potencies in part due to inadequate drug stability and inefficiencies in cancer-selective drug delivery. As such, innovative strategies regarding drug design and delivery are of utmost importance to achieve improved treatment results. With our current study, we aimed at exploring the groundwork for a two-stage targeting concept, which is based on the intrinsic tumor homing capacity of mesenchymal stem cells (MSCs) as cellular drug factories for the *in situ* production of our newly designed and biomarker-targeted TRAIL-based TR3 therapeutics. Since MSCs are primary cells, capable *in vitro* of only a limited number of cell divisions, identification of suitable strategies for their efficient genetic manipulation is of critical importance. We chose adenoviral (Ad) vectors as a transduction vehicle due to its ability to infect dividing and non-dividing cells and because of their limited restrictions regarding the packaging capacity of their genetic payload. In order to enhance the transduction efficacy of MSCs using Ad5 wild-type-based vectors, we tested a variety of fiber knob modifications on a panel of patient-derived MSC lines established from adipose tissue. We identified Ad5pK7, an Ad5 vector containing a polylysine fiber knob modification, exhibiting the highest transduction rates across a panel of 16 patient-derived MSC lines. We further demonstrated that MSCs could be efficiently transduced with an Ad5pK7 vector containing membrane-anchored and secreted TR3 expression units, including the MUC16 (CA125)-targeted variant Meso64-TR3. In both *in vitro and in vivo* experiments, MSC-derived Meso64-TR3 was far more potent on MUC16-expressing ovarian cancer compared to its non-targeted TR3 counterpart. Our findings thus provide the foundation to initiate further preclinical investigations on MSC-mediated treatment options in ovarian cancer using biomarker-targeted TR3-based biologics.

## Introduction

Ovarian cancer causes more deaths than any other cancer of the female reproductive tract, and at best, 5-year survival rates are approximately 46% [1, 2]. Therefore, the need for novel anticancer strategies is of paramount importance. Efficient delivery of novel systemically administered cancer therapeutics remains an important challenge in drug development, especially within the field of gynecologic oncology.

Tumor necrosis factor-related apoptosis-inducing ligand (TRAIL) represents a promising anti-cancer therapeutic due to its ability to induce apoptosis upon binding to its death receptors DR4 and DR5 [3–8]. Since the first report describing TRAIL in 1995 [5], the majority of research has explored this molecule as an anti-cancer therapeutic, capitalizing on its ability to selectively induce apoptosis in a broad range of tumor cell lines with minimal effect on normal cells [4, 5, 9]. Unfortunately, major challenges utilizing conventional TRAIL in clinical practice include possible off-target toxicity in the liver and brain [10, 11] and rapid clearance from the body with a half-life of approximately one hour [12], thus requiring repeated injections to maintain high enough concentrations to achieve potential therapeutic responses [13].

The need for a more stable therapeutic compound with efficient and selective tumor cell elimination led us to explore architectural modifications of the TRAIL molecule itself. Recombinant and endogenous TRAIL require trimerization in order to gain functional activity, but are prone to rapid inactivation via trimer dissociation. Therefore, we redesigned recombinant TRAIL by creating a head-to-tail fusion protein of its three protomers, designated TR3, characterized by high stability and a unique stoichiometry with only one amino-terminus and one carboxyl-terminus [14]. We also explored several downstream modifications of the TR3 drug platform. Taking advantage of the high-affinity interaction between mesothelin and the MUC16 biomarker located on ovarian cancer cell membranes [15], we designed a mesothelin/TR3 fusion protein [16], and subsequently a more potent and stabilized truncation variant, Meso64TR3 [17]. Compared to non-targeted, parental TR3, such membrane conversion resulted in far more death receptor signaling and apoptosis induction [16, 18, 19]. Furthermore, the unique stoichiometry of TR3 allowed us to modify the carboxyl-terminus and generate functional transmembrane- and glycosylphosphatidylinositol (GPI)-anchored variants with and without spacer domains, e.g. TR3GPI and TR3DAF, respectively [19].

Combining these TR3 modifications with an efficient cellular delivery system to enhance tumor specificity has not yet been explored. The tumor-homing capacity of mesenchymal stem cells (MSCs) offer exciting avenues to harness these cells as efficient, drug delivery vehicles in combination with their high gene transduction efficiency and ability to evade immune recognition and elimination [20]. MSCs are isolated from bone marrow or adipose tissue and have an inherent ability to migrate to and engraft both the primary tumors and metastatic sites [21–27], thereby serving as an attractive cellular vehicle to enhance ovarian cancer therapy. A two-stage targeting strategy using MSCs “armed” with selective TR3 therapeutics warrants investigation; however, the feasibility of producing such cellular carriers remain to be explored.

Therefore, we developed a transduction strategy based on a DNA virus, adenovirus (Ad), known to have the ability to transduce dividing and non-dividing cells with a broad host tropism [23–27]. Primary attachment of the virus is mediated by the knob region of the fiber, which binds to the coxsackie adenovirus receptor (CAR) [28], followed by internalization into the host cell [29]. Hence, gene delivery is strongly dependent on the CAR expression levels of the target cells. Unfortunately, this is highly variable in human MSCs resulting in unpredictable and highly variable transduction efficiencies ranging from 20–40% [30, 31]. Therefore, we initially assessed the efficiency of Ad5-mediated transduction of both membrane-anchored and soluble TR3 variants in CHO-CAR cells, Chinese Hamster Ovary cells that ectopically express human CAR, and tested the bioactivity of the respective drug products functionally. We then focused our attention to investigating the Ad5 delivery platform in adipose-derived human MSCs. We therefore set out to isolate and culture MSCs from human adipose tissue and then compare transduction efficiencies of MSCs using different fiber-modified Ad5 vectors in order to optimize gene transfer rates. We finally tested MSC-derived TR3 variants for functional activity in MUC16-deficient and MUC16-positive cancer cells *in vitr*o to test our hypothesis that MSC-derived supernatant containing Meso64TR3 has enhanced activity profile against MUC16-positive OVCAR3 cells relative to parental TR3 and Ad5 control. Preliminary data did in fact support the idea that Ad5pK7-infected adipose-derived MSCs are also functional *in vivo*.

## Materials and methods

### Cells and reagents

Isolation and culture of MSCs were obtained from human adipose tissue. All patient-derived tissue was obtained in full compliance with and approval of the institutional review board of Washington University School of Medicine (IRB ID# 201108117). Approximately 1 gram of subcutaneous adipose tissue was obtained from patients operated on by the gynecologic oncology division at Barnes-Jewish Hospital. In agreement with previous report Crespo-Diaz et al [32], the tissue was minced with a surgical scalpel for 5 minutes and incubated at 37°C in a petri dish containing 0.075% collagenase type 1 (Worthington Biochemical, Lakewood, NJ) for 90 minutes. Digested tissue was then transferred to a 50 mL conical tube with 30 mL of MSC media and centrifuged at 400 x g for 5 minutes. The supernatant was aspirated and the pellet was resuspended in 12 mL of MSC media (advanced MEM with 5% PLT Max [Mill Creek Life Sciences], 2 mM L-glutamine, 100 U/mL penicillin and 100 mg/mL streptomycin). All other cell lines used in the experiments were obtained from the American Type Culture Collection (ATCC, Manassas, VA). The human T cell line Jurkat was maintained in Roswell Park Memorial Institute (RPMI1640) medium (Invitrogen), supplemented with 10% FCS, L-glutamine and penicillin/streptomycin. CHO-CAR cells were maintained in Ham’s F12 medium (Gibco, Life Technologies, Grand Island, NY), supplemented with 10% FCS, L-glutamine and penicillin/streptomycin [28].

For Western blot analyses, anti-human TRAIL pAb (rabbit) was obtained from Peprotech (Rocky Hill, NJ). Signal detection was achieved with goat anti-rabbit HRP-conjugated secondary antibodies (Santa Cruz Biotechnology, Santa Cruz, CA). A molecular weight marker (Precision Plus Protein Western C Standards and Precision Protein StrepTactin-HRP Conjugate), was obtained from Bio-RAD (Hercules, CA). TRAIL detection for flow cytometry applications was performed using a function-blocking mouse mAb (clone 2E5) purchased from Abcam (Cambridge, MA). For fluorescence-activated cell sorting (FACS) analyses, secondary anti-mouse pAb (IgG) (FITC/PE conjugated) was obtained from Sigma-Aldrich (St. Louis, MO). Z-VAD-FMK was obtained from Enzo Life Sciences (Farmingdale, NY). Custom oligonucleotides were purchased from Integrated DNA Technologies (IDT, Coralville, IA).

### Expression plasmids

The basic TR3 expression plasmid was described previously [14]. The membrane anchored TR3 variants TR3-GPI and TR3-DAF [19], and secreted forms MesoTR3 and Meso64TR3 [16, 17], have been described previously.

### Generation of recombinant adenovirus vectors

To generate replication incompetent Ad vectors for TR3eYFP, TR3GPIeYFP, TR3DAFeYFP, and Meso64TR3eYFP, the shuttle plasmids containing the respective genes controlled by a strong Cytomegalovirus (CMV) promoter were constructed. Then we incorporated the CMV-driven expression cassettes in place of the early E1 region by homologous recombination in *E*. coli BJ5183 with a pAdEasy-1 plasmid, which contains the Ad5 genome devoid of the E3 genes [33].

The following describes generation of recombinant Ad vectors for membrane anchored TR3 variants. The plasmids carrying Ad5-TR3GPIeYFP and Ad5-TR3DAFeYFP genomes were validated by polymerase chain reaction (PCR), restriction analysis, and partial sequencing, linearized with *Pac*I to release the inverted terminal repeats of the viral genomic DNA and used to transfect HEK293 cells. The rescued Ad5-TR3GPIeYFP and Ad5-TR3DAFeYFP vectors were propagated using 911 cells [34], purified by centrifugation on CsCl gradients according to standard protocols, dialyzed against phosphate-buffered saline (PBS) (8 mM Na_2_HPO_4_, 2 mM KH_2_PO_4_ [pH 7.4], 137 mM NaCl, 2.7 mM KCl]) containing 10% glycerol, and stored at −80°C until further use. The titers of physical viral particles (v.p.) were determined by methods described by Maizel *et al*. [35] and calculated as 1.95 × 10^12^/mL for Ad5-TR3GPIeYFP and 2.25 × 10^12^/mL for Ad5-TR3DAFeYFP, respectively. The same strategy was used to generate the recombinant Ad vectors for the secreted variants TR3 and Meso64TR3.

To construct infectivity-enhanced Ad vectors for MSC infection we employed the fiber protein modification relying on the C-terminal incorporation of polylysine motif (pK7) containing seven lysine residues as previously described [36]. The generation of infectivity-enhanced Ad5 vectors was carried out using viral genome encoding fiber-K7 protein for homologous recombination with corresponding shuttle plasmid essentially as described above [37]. All vectors contained a reporter gene(s), either a green (GFP) or yellow (YFP) fluorescent protein in the E1 region of the Ad5. The titers were determined as 4.3 × 10^11^ v.p./mL for Ad5K7-TR3eYFP and 6.3 × 10^11^ v.p./mL for Ad5K7-Meso64TR3eYFP, respectively.

### Gene transfer assay

Four different low passage MSC cell lines (Patient #5, 6, 8 and 9) derived from human adipose tissue were plated in 24-well plate, each well containing 16,000 cells in a total volume of 500 μl. The monolayers of MSCs were infected at the MOI of 5000 v.p./cell with the following Ad5-based vector derivatives described previously [37]. Ad5RGD contains the arginine-glycine-aspartate (RGD)-4C motif in the HI loop of the fiber knob. Ad5pK7 contains a pK7 motif at the C-terminus of the fiber protein. Ad5RGDpK7 has both RGD-4C and pK7 motif. Ad5/3 has the fiber knob domain replaced with its counterpart from serotype 3, which binds the cellular receptor different from human coxsackievirus and adenovirus receptor (CAR) [38]. Ad5/PK4 has fiber knob replaced with the knob domain derived from the NADC-1 strain of porcine Ad type 4, which contains the tandem carbohydrate binding domains [39]. The gene transfer efficiencies achieved by the fiber-modified Ad vectors were compared to Ad5 control using the GFP reporter encoded by each virus and epifluorescence microscopy to detect fluorescent infected cells 48 hours postinfection. We used the firefly luciferase encoded by each virus and a luciferase assay (Promega, USA) to quantify the levels of gene transfer demonstrated by fiber-modified Ad vectors with respect to control Ad5 vector.

### Adenovirus transduction efficiency

Seven different multiplicities of infections (MOIs) were tested in each of the three adenovirus constructs (Ad5-eYFP, Ad5-TR3eYFP, and Ad5-Meso64TR3eYFP). Using a 24-well format, 1.7x10^5^ CHO-CAR cells/well were infected with Ad5-eYFP (MOI 1000), Ad5-TR3GPIeYFP (MOI 5000), Ad5-TR3DAFeY (MOI 8750) and treated for 7 hours. To assess the percentage of YFP positive populations, cells were washed and harvested non-enzymatically (EDTA) 2 days after the transduction. The cells were then submitted to flow cytometry (FACSCalibur, BD Biosciences, San Jose, CA).

### Immunoblotting

Samples (cell lysates or transfection/infection supernatants) were submitted to 10% SDS-PAGE and transferred onto a nitrocellulose membrane. After blocking with dry milk, the membranes were incubated with the respective primary antibodies (anti-human TRAIL pAb, anti-human mesothelin mAb or anti-FLAG mAb), followed by HRP-conjugated secondary antibodies (anti-rabbit or anti-mouse) and developed with Immunstar Western C kit (Bio-Rad, Hercules, CA) using the Chemidoc XRS plus Imaging system (Bio-Rad).

### Flow cytometry

MSC marker expression was determined by flow cytometry. MSCs grown in T75 flasks were washed with PBS, harvested by trypsin and resuspended in PBS containing 1% FBS. 60,000 cells/ml were then aliquoted per Eppendorf tube, centrifuged at 1200 rpm x 5 minutes, and incubated in 100 μL of a 1:20 dilution of primary monoclonal antibodies directed against CD90 and CD 166, coupled to phycoerythrin (PE); CD105 and CD49d, coupled to Allophycocyanin (APC); or directed against CD73, coupled to fluorescein isothiocyanate (FITC). Cells were incubated for 30 minutes on ice. After cells were washed with 1 mL of FACS buffer, 1 μg/mL propidium iodide (Sigma Chemical Co.) was added to sort out dead cells from the sample. Cells were then analyzed immediately by flow cytometry (FACSCalibur, BD Biosciences, San Jose, CA) along with PE-, APC-, FITC-isotype controls. Data acquisition was done on a FACSCalibur flow cytometer (BD Biosciences, San Jose, CA) and data were analyzed using FlowJo software (Version 7.6.5, Tree Star, Ashland, OR).

### Cell death determinations

The killing capacity of the secreted TR3 variants (TR3 and Meso64TR3) produced from CHO-CARs and MSCs were assessed using a cell viability assay. Jurkat and OVCAR-3 cells were seeded into 96-well plates at the respective optimal densities (5x10^4^ and 1x10^4^ cells respectively). Treatment was initiated the following day with supernatant from TR3-infected CHO-CARs and MSCs and cell death was determined 18 hours after treatment using CellTiterGlo Luminescent Viability Assay following the manufacturer’s instructions (Promega Madison, WI). Data were recorded using a MultiDetection Microplate Reader (Synergy HT, BioTek, Winooski, VT).

The killing capacity of our novel, membrane-anchored TR3 (TR3GPI) was assessed employing a morphology-based FACS assay using Jurkat reporter cells. Unless otherwise stated, the simplified protocol was employed to compare the cell killing activities of the various TRAIL forms. Data acquisition was done on a FACScan flow cytometer (Becton & Dickinson, Bedford, Maryland). The data were analyzed with FlowJo software (Version 7.2.5, Tree Star, Ashland, Oregon). Cell viability was determined by CellTiter-Glo (Promega) according to the manufacturer’s instructions. Data were recorded with a luminescence plate reader (Molecular Devices, SpectraMAX-Gemini, SoftMax Version 5 software, Sunnyvale, California).

### Drug treatment in the presence of apoptosis blockade

Z-VAD-FMK (carbobenzoxy-valyl-alanyl-aspartyl-[O-methyl]fluoromethylketone), a cell-permeant, irreversible pan-caspase inhibitor, was used to confirm involvement of the extrinsic arm of apoptosis induced by Ad-produced TR3 biologics. Jurkat cells, seeded into 96-well plates at 5 x 10^4^ cells per well, were treated with the secreted TR3 variants (TR3 and Meso64TR3) produced from CHO-CARs infected with similar efficacy with the respective adenoviruses, Ad5-TR3eYFP and Ad5-Meso64TR3eYFP. Cell death in the absence and presence of 1 μM Z-VAD-FMK was determined 18 hours after treatment using the CellTiterGlo Luminescent Viability Assay as described above (Promega). Data were recorded using a MultiDetection Microplate Reader (Synergy HT, BioTek).

### Statistical analyses

Treatment efficiency of *in vitro* killing assays are presented as means ± SEM. Statistical significance is defined as *P* < 0.05 and was calculated employing analysis of variance (one-way ANOVA, Tukey’s Multiple Comparison Test) and the Student’s *t-test* (unpaired) as indicated using GraphPad Prism (V 6.04) software.

## Results

### Design and structural features of adenovirally transduced TR3 variants from mammalian cells

**Fig 1:**
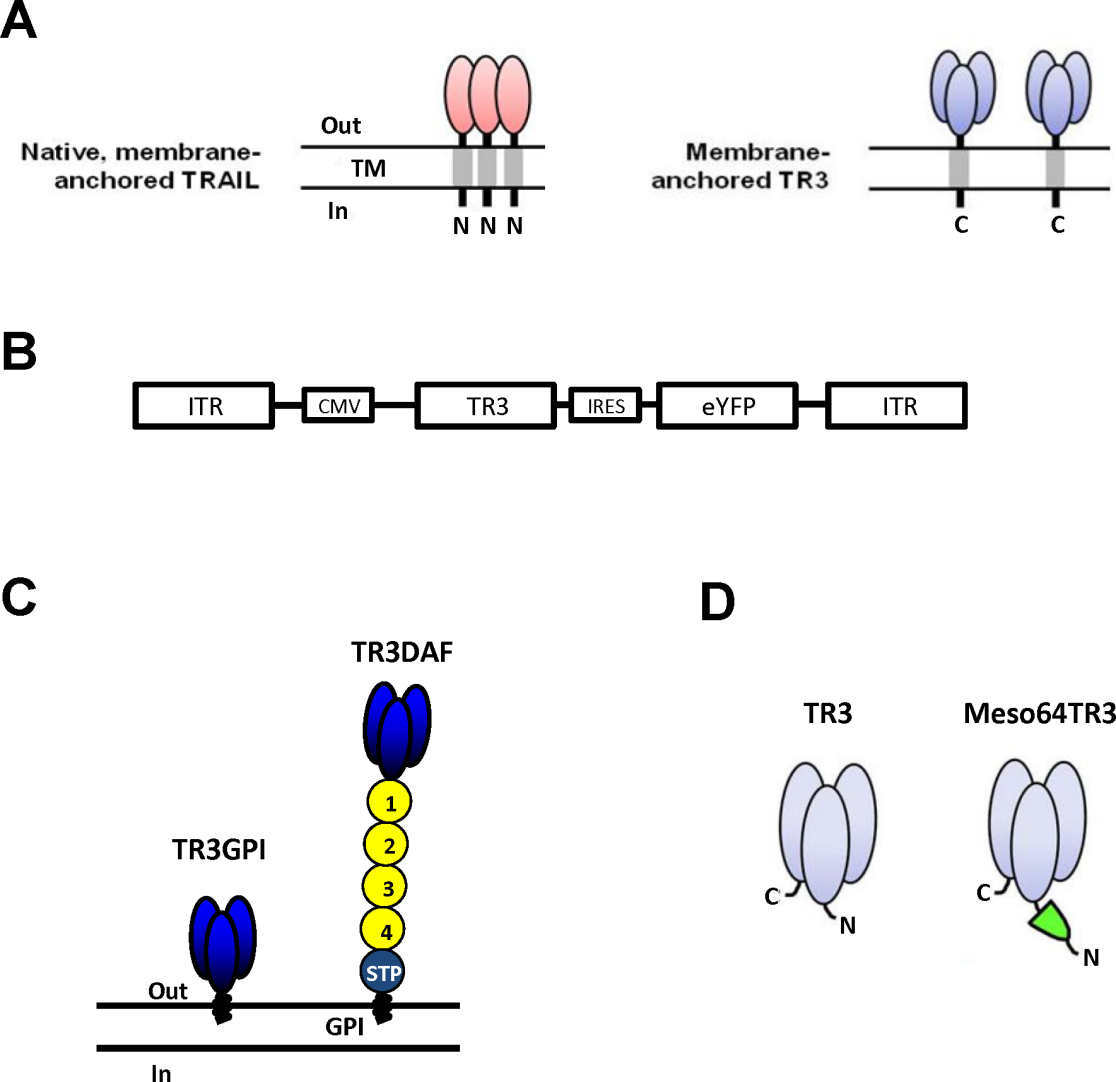
Schematic representation of native TRAIL and recombinant TR3 variants used in this study. **(A)** Illustration of the key structural features of wild-type, native TRAIL and a recombinant TR3 form anchored to the membrane via carboxyl-terminal incorporation of a transmembrane domain (TR3-TM). Please note the vastly different domain architecture/stoichiometry of the trimers between TRAIL (1:1) and TR3-TM (1:3). **(B)** Bicistronic adenoviral vector design in which TR3 expression correlates with the expression level of yellow fluorescent protein (eYFP) via Internal Ribosome Entry Site (IRES)-mediated cotranslation of the mRNA. **(C)** Schematic representation of two membrane-anchored TR3 variants are depicted: TR3GPI, in which the GPI-encoding sequence of human DAF servers as anchors signal and TR3DAF, in which the entire mature form of human DAF serves as an anchor unit. The short consensus repeats (SCRs) of DAF are indicated (1–4) and serve as a molecular spacer relative to the plasma membrane. **(D)** Secreted variants TR3 (parental) and Meso64TR3 are shown, the latter representing a MUC16-targeted TR3 trimer, in which only the 64 amino-terminal amino acids of mesothelin were used as delivery moiety to the MUC16 biomarker (including an N-terminal FLAG tag, not shown).

Endogenous, native TRAIL self-assembles into three non-covalently associated homotrimers at the plasma cell membrane to become biologically active with the amino-termini (N-termini) of the individual protomers pointing to the cytoplasm of the cell (Fig 1A, left panel, type-II membrane protein). In contrast, membrane-anchored and fully bioactive TR3 trimers require only a single polypeptide chain and are inserted into the plasma membrane as classical type-I membrane proteins, with their carboxyl-termini (C-termini) facing the cytoplasm (Fig 1A, right panel). The resulting stoichiometry with only one N- and one C-terminus per trimer represents a characteristic feature of all TR3-based biologics, whereas trimers based on wild-type TRAIL contain three N- and C-termini each, respectively. In general, all recombinant TR3 variants can be produced in both secreted and membrane-anchored forms.

For consistency reasons, all TR3 variants described in this study were part of a bicistronic expression cassette [19, 40, 41] in conjunction with a yellow fluorescent protein (eYFP) as a marker for monitoring transduction efficiencies (Fig 1B). Our membrane-anchored TR3 variants included: 1) TR3-GPI, which anchors to the membrane via a glycosyl-phosphatidylinositol (GPI)-encoding signal derived from human decay-accelerating factor (DAF), and 2) TR3-DAF, which anchors to the membrane using the entire mature form of human DAF and contains 4 short consensus repeats in addition to an elongated stalk region (Fig 1C). Furthermore, in order to study soluble TR3 variants, we generated Ad5-based vectors encoding non-targeted TR3 and Meso64TR3 (Fig 1D and Ref. [17]), the latter suitable for the site-specific delivery to the biomarker MUC16 (CA125) overexpressed by the majority of epithelial ovarian cancers.

### Preserved bioactivity of membrane-anchored TR3 variants produced from mammalian cells

**Fig 2:**
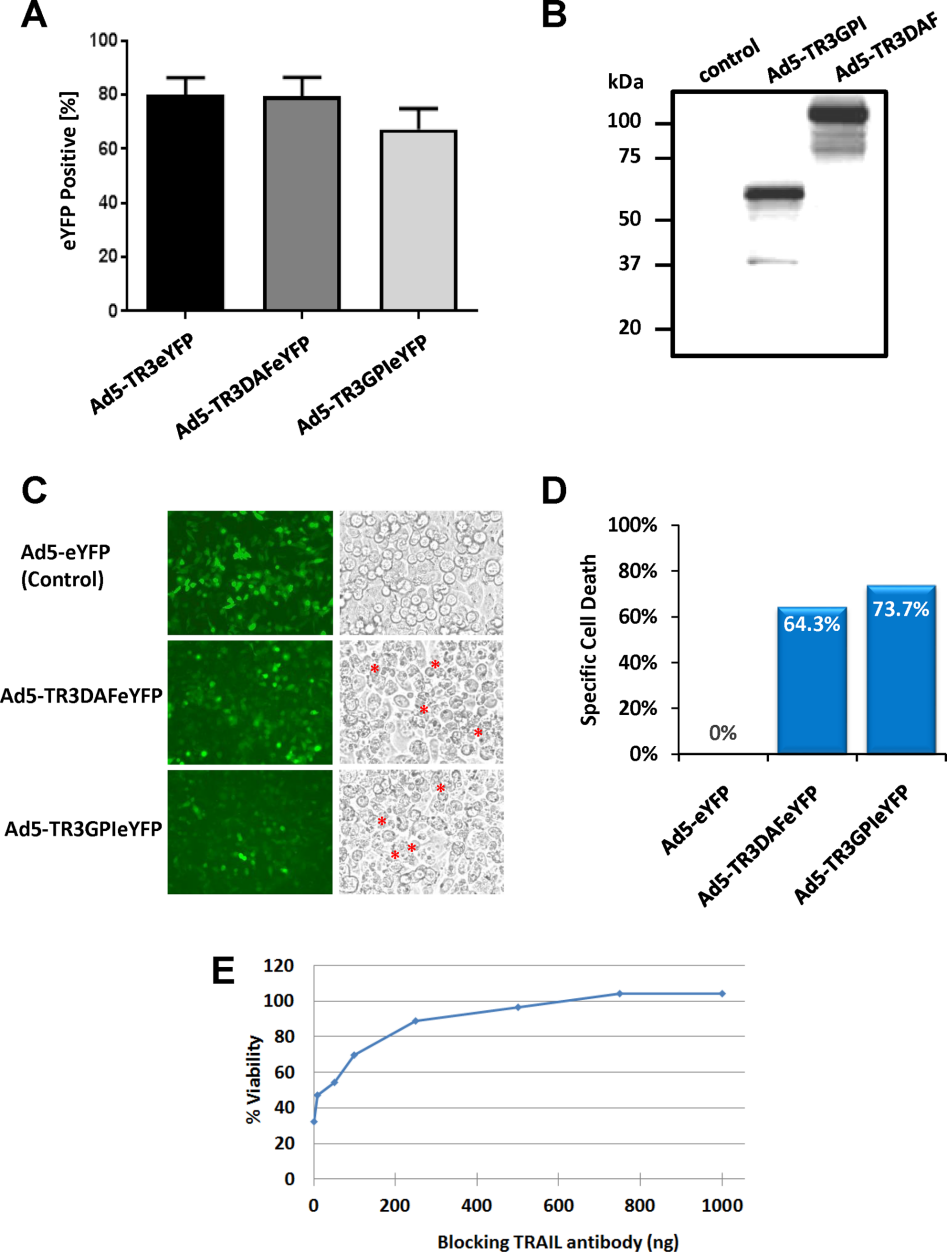
CHO-CAR cells expressing membrane-anchored TR3 variants induce cell-cell contact-dependent target cell death. **(A)** CHO-CAR cells were infected with the indicated Ad5-based viruses using MOIs that were determined to achieve equivalent transduction efficiencies during prior titration experiments. Infection of the cells with Ad5-eYFP (MOI 1000), Ad5-TR3GPIeYFP (MOI 5000) and Ad5-TR3DAFeYFP (MOI 8750) did indeed result in comparable transduction efficacies gauged on the proportion of YFP-positive cells using flow cytometry. **(B)** Western Blot analysis of infected CHO-CAR cell lysates confirmed the molecular weights of TR3GPI (~65 kDa) and TR3DAF (~130 kDa). **(C)** Functional TR3 activity was assessed employing a co-culture experiment with Jurkat suspension cells. Two hours later, and compared to the control (GFP), large numbers of apoptotic bodies were noted only on cells expressing the membrane-anchored TR3 variants (asterisks), indicative of widespread apoptosis induction. **(D)** Graphic representation of the overlay assay shown in (C), with specific cell death of ~64% mediated by expression of TR3GPIeYFP and ~74% by TR3DAFeYFP on the CHO-CAR effector cells determined by FACS analysis. **(E)** Blocking experiment using anti-TRAIL antibody to demonstrate mechanism of cell-cell contact-dependent mechanism of cell death. The contact-dependent killing capacity of TR3GPI-expresssing CHO-CARs (Ad5-TR3GPIeYFP) co-cultured with Jurkat suspension cells was dose-dependent and was completely abolished at the highest concentration of TRAIL-blocking antibody (> 600 ng).

In an initial attempt to assess if Ad5-based vectors are capable of delivering full length TR3-containing genetic information, CHO-CAR cells were infected with the membrane-anchored TR3 variants Ad5-TR3GPIeYFP and Ad5-TR3DAFeYFP, as well as control vector Ad5-eYFP. Based on a number of pilot experiments (not shown), the following conditions were used: Ad5eYFP—MOI 1000; Ad5-TR3DAFeYFP—MOI 8750; Ad5-TR3GPIeYFP—MOI 5000 and resulted in comparable transduction rates for all three viruses between 70% and 80% gauged by the ratio of YFP-positive cells (Fig 2A). Western Blot analysis of infected CHO-CAR cell lysates confirmed the molecular weights of TR3GPI and TR3DAF of ~61 kDa and ~130 kDa, respectively (Fig 2B). Cell surface stain against the TR3 epitope (anti-TRAIL mAb) confirmed the membrane localization of TR3GPI and TR3DAF (S1 Fig).

In order to assess the functional activity of our membrane-anchored TR3 variants, we conducted a series of cell death experiments using an overlay co-culture configuration with Jurkat suspension cells. Twenty-four hours post infection, using the above described conditions to achieve equivalent infection efficacies (Fig 2C, left panel), the CHO-CAR cells were co-cultured with TRAIL-sensitive Jurkat cells and functional activity was assessed for each membrane-anchored TR3 variant. CHO-CAR cells infected with the YFP control virus did not cause any detectable cell death (Fig 2C, control), while TR3-expressing CHO-CAR cells caused substantial cell death as visualized by the appearance of apoptotic bodies (Fig 2C, asterisks). A quantitative analysis by flow cytometry of the same overlay assay confirmed a substantial specific cell death mediated by expression of TR3DAF (64%) and TR3GPI (74%) on the CHO-CAR cell membrane (Fig 2D).

In a next step, we sought to validate that the cell death was indeed caused by functional TR3 expression on the surface of the effector cells [14] employing a blocking anti-TRAIL monoclonal antibody (mAb) during the co-culture experiment. We found that increasing concentrations of anti-TRAIL mAb reduced the killing capacity of TR3GPI-expressing CHO-CAR cells in a dose-dependent manner, resulting in a complete drug inhibition at a saturating antibody concentration (Fig 2E).

### Preserved bioactivity of secreted TR3 variants produced from mammalian CHO cells via adenoviral infection

**Fig 3:**
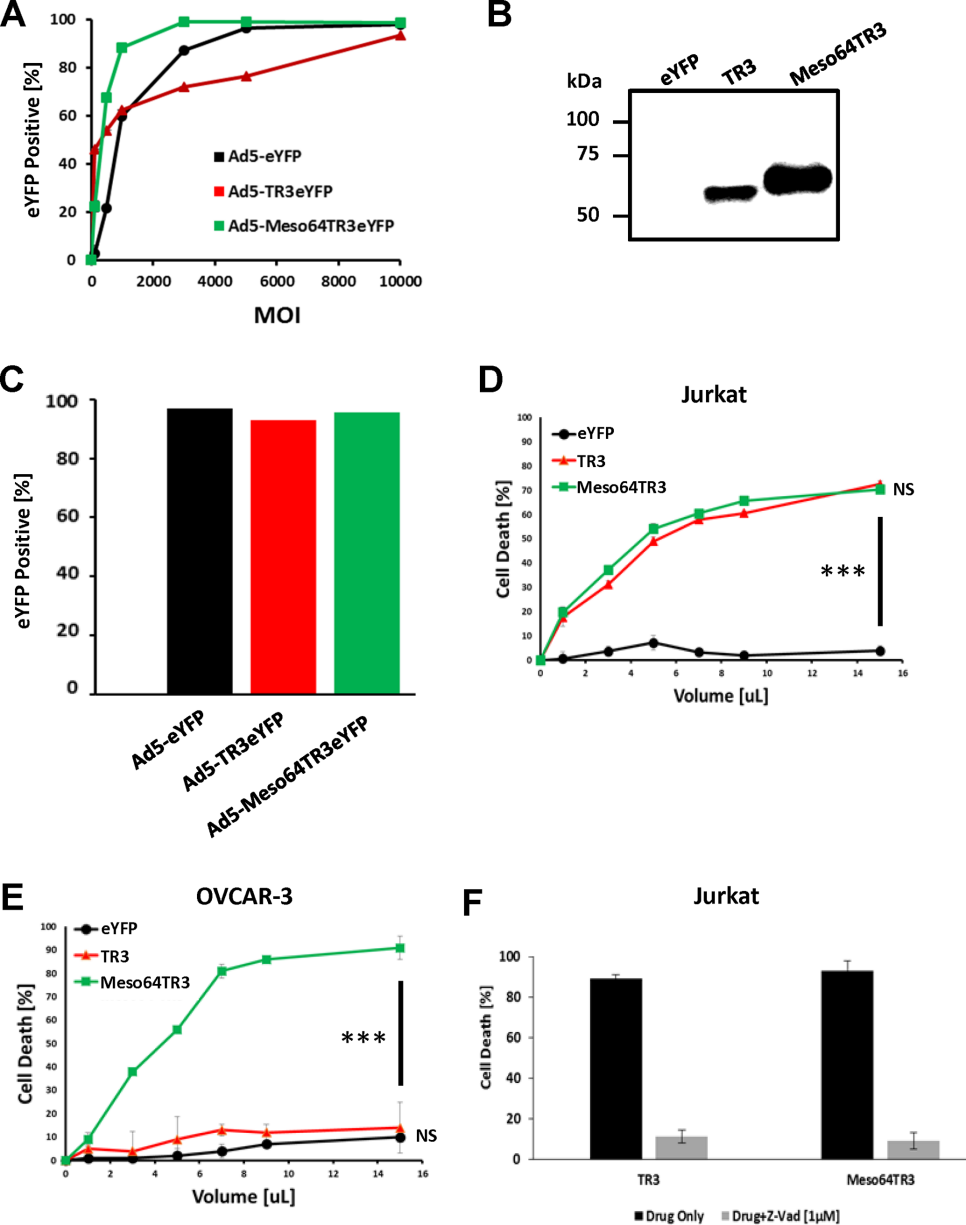
Adenovirus transduction of CHO-CAR cells to produce soluble, biologically active TR3 variants. **(A)** In order to confirm efficient virus production, CHO-CAR cells were infected with increasing multiplicities of infections. The eYFP signal was used as an indicator of expression level of the respective biologics. All viruses transduced the reporter cell in a dose-dependent fashion. **(B)** Western Blot analysis of supernatant from CHO-CAR-infected cells (MOI 10000) confirmed production and molecular weights of TR3 and Meso64TR3 at ~61 kDa and ~65 kDa, respectively. **(C)** In order to produce secreted TR3 drugs for functional activity testing, plateau-reaching MOIs were used for the respective virus preparation. These were subsequently confirmed by flow cytometry to ensure that infection rates resulted in equivalent production of the respective biologics (Ad5-eYFP, MOI 5000 [control]; Ad5-TR3eYFP, MOI 10000; Ad5-Meso64TR3eYFP, MOI 3000). **(D)** Cell killing profiles of TR3 and Meso64TR3 at increasing drug volumes were established on MUC16-deficient T cell leukemia cell line Jurkat. Supernatant of cells infected with Ad5-eYFP was used as a control. Please note that both drugs induced a dose-dependent cell death overlapping response curves, consistent with their known activity profiles. ***, P < 0.0002. **(E)** The same cell death determination as in (D) using identical drug volumes was performed in MUC16-positive OVCAR3 cells. Please note the much enhanced activity profile of the MUC16-targeted cancer drug Meso64TR3 relative to parental TR3. NS, not significant; ***, P < 0.0002. **(F)** In order to confirm extrinsic pathway involvement as the cause cell death induction, Jurkat cells were treated with a constant amount of TR3 and Meso64TR3 in the presence of Z-VAD-FMK, a pan-caspase inhibitor. Z-VAD-FMK completely eliminated the killing capacity of both biologics. ***, P < 0.0002.

We next explored the possibility of producing secreted TR3 variants using Ad5-based vectors, including TR3 and the MUC16-targeted variant Meso64TR3. We therefore infected CHO-CAR cells with Ad5-TR3eYFP, Ad5-Meso64TR3eYFP, and YFP control virus (Ad5-eYFP), at increasing MOIs. Gauged on the expression of the fluorescent marker, all three viruses transduced the target cells in a dose-dependent manner, reaching a plateau at MOIs of 3000 (Meso64TR3), 5000 (YFP control) and 10000 (TR3), respectively (Fig 3A). Western Blot analysis of supernatant from CHO-CAR-infected cells confirmed the molecular weights of TR3 (~61 kDa) and Meso64TR3 (~65 kDa) (Fig 3B). In an effort to obtain equivalent concentrations of the secreted proteins for functional testing, we infected CHO-CAR cells with the plateau-reaching MOIs determined by virus titration as shown above (Fig 3A). This resulted in comparable transduction efficiencies of greater than 95% for all three viruses as confirmed by flow cytometry at the time of drug collection (Fig 3C).

Since the YFP reporter was used to monitor transduction efficiencies, we wondered if similar transduction efficacies would correlate well with the production level, i.e. functional activity of the respective TR3 drugs. We therefore assessed the bioactivity profiles of our secreted TR3 variants on MUC16-deficient Jurkat reporter cells. It turned out that, in contrast to the biologically inactive eYFP control supernatant, both TR3 and Meso64TR3 showed nearly identical activity profiles, indicative of containing similar drug amounts (p<0.0002) (Fig 3D). These data are consistent with the notion that the bioactivity of TR3 and Meso64TR3 have been determined to be equivalent on MUC16-deficient cancer cells [17]. Based on our prior work [17] in which Meso64TR3, produced in HEK293T cells, caused very strong cytotoxicities in MUC16-expressing cancer cells (ovarian and pancreatic cancer), we tested whether Meso64TR3 produced from CHO-CAR cells retained its targeted bioactivity on MUC16-positive ovarian cancer cells OVCAR3. Therefore, 24 hours post-infection with Ad5-Meso64TR3eYFP, Ad5-TR3eYFP, and Ad5-eYFP, the CHO-CAR supernatants were functionally tested on OVCAR3 cells. Meso64TR3 retained its strong bioactivity, while TR3 was no more effective than the eYFP control supernatant (Fig 3E). We also confirmed the underlying cell death mechanism by conducting cell viability assays in the presence of Z-VAD-FMK, a powerful and irreversible pan-caspase inhibitor to block the TR3-induced extrinsic death pathway. Indeed, using MUC16-deficicent Jurkat reporter cells, the death-inducing capacity of both TR3 therapeutics was substantially reduced in the presence of Z-VAD-FMK from 90% (bioactivity of both drugs) to 10% (Fig 3F), similar to the cell death rate of a Jurkat cell culture at steady-state (not shown).

### Generation, characterization, and genetic engineering of MSCs derived from human adipose tissues

**Fig 4:**
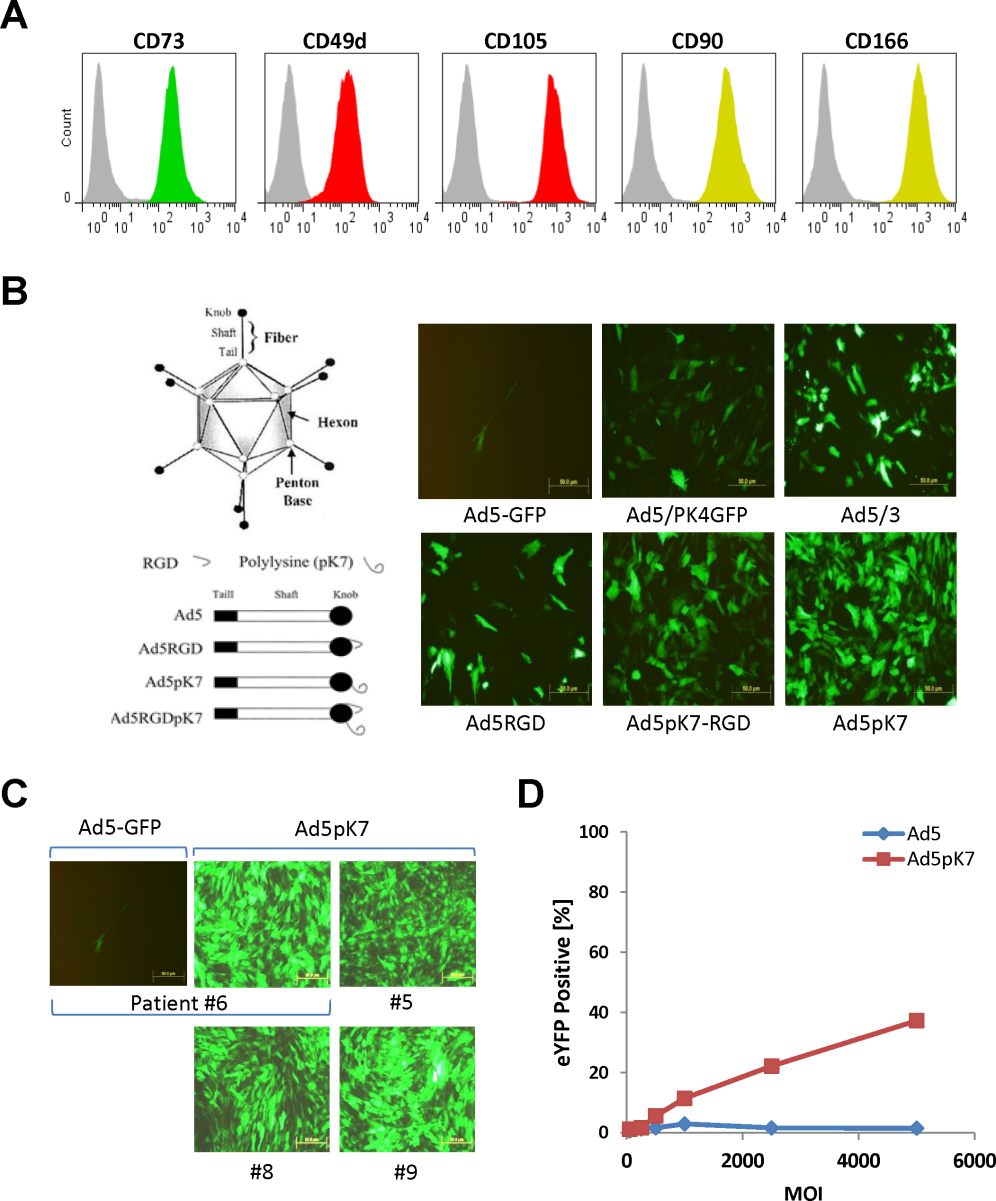
Exploring human MSCs to serve as cellular carriers for TR3-based cancer therapy. **(A)** In a first characterization step, we aimed at verifying a set of surface markers present on undifferentiated human adipose-derived mesenchymal stem cells by flow cytometry. MSCs from an individual donor were stained with monoclonal antibodies directed against CD90, CD 166, CD105, CD49d and CD73. Based on the respective, homogeneous biomarker expression profiles, we could confirm that all of our generated stable cell lines were indeed MSCs. **(B)** A panel of Ad5 vectors was evaluated for gene transfer efficiency on human MSCs. Among the variants tested were: Ad5-GFP (wild-type), which expressed the marker luciferase and green-fluorescence protein; Ad5/PK4GFP, an Ad5-based vector containing a pK4 motif at the C-terminus; Ad5/3, a variant featuring a chimeric fiber protein knob domain derived from Ad3; Ad5RGD, an Ad5 vector containing a heterologous RGD motif in the HI loop of the fiber knob; Ad5pk7-RGD, a double-modified Ad5 vector containing arginine-glycine-aspartate (RGD) motif in the HI loop and a pK7 motif at the C-terminus; and Ad5pK7 is an Ad5 vector containing a pK7 motif at the C-terminus (courtesy Contreras et al 2003). This screening effort resulted in the identification of Ad5pK7 giving rise to the highest transduction efficacy among all other tested Ad5 variants using a representative and randomly chosen MSC line. **(C)** In order to demonstrate broader applicability of the pK7 fiber knob as a more universal delivery vehicle to transduce human MSCs, we tested additional adipose-derived MSC lines. Compared to an Ad5 wild-type control, Ad5pK7 was capable of infecting a broad range of MSC lines with nearly 100% transduction efficiency. **(D)** A dose-escalation curve confirms the enhanced infectivity rate of Ad5pK7 compared to control Ad5.

After we demonstrated that CHO-CAR cells could be efficiently transduced with Ad5 wild-type-based vectors and secrete highly functional, biomarker-targeted TR3-based therapeutics, we focused on the secondary study objective: the generation of human MSCs derived from adipose tissue with the ultimate goal of “arming” our cellular vehicles with TR3-based cancer therapeutics. Therefore, we established 16 stable MSC lines from patient-derived adipose tissues obtained from 12 newly diagnosed gynecologic oncology patients (four ovarian, four uterine and three fallopian tube, one primary peritoneal carcinoma), one patient with recurrent colon cancer, and three benign patients undergoing surgery via an exploratory laparotomy. After two passages *in vitro*, MSCs represented a homogenous population of undifferentiated fibroblast-shaped cells. As a means to verify cellular authenticity, we performed a flow cytometry-based characterization of MSC-specific cell surface markers, including CD49d (integrin α4 chain), CD73 (ecto-5`-nucleotidase), CD90 (Thy-1), CD105 (Endoglin), and CD166 (CD6 ligand, activated leukocyte cell adhesion molecule [ALCAM]) [31, 42, 43]. All of the 16, adipose-derived cell lines showed the anticipated FACS profiles with regard to their in signal intensities (copy numbers) and shapes (homogeneous protein expression patterns) for all these surface markers, consistent with a human MSC expression profile (Fig 4A, FACS profiles of a representative MSC cell line shown).

Based on published reports, we anticipated transduction rates of human MSCs with Ad5-based vectors to range between 20% and 40% [30, 31]. Unfortunately, in our hands, Ad5 transduction efficiencies across a panel of human adipose-derived MSCs was less than 5% (not shown). Given these unexpected results, we screened a panel of fiber-modified Ad5 vectors on a randomly chosen human, adipose-derived MSC line in an effort to identify a serotype that gave rise to the highest transduction efficiency using fluorescent protein expression as a readout. Compared to an Ad5 control vector, Ad5pK7, which contains a C-terminal pK7 motif, effectively transduced our selected line of MSCs and outperformed the other Ad variants tested (Fig 4B). To rule out that the initial high transduction rate was not limited to a single MSC line, Ad5pK7 was tested against additional, randomly chosen adipose-derived MSC lines, all of which were consistently infected with a nearly 100% rate (Fig 4C). A formal and quantitative comparison between the Ad5pK7 variant and its wild-type Ad5 counterpart demonstrated a dose-dependent increase in transduction efficacy for the former, while the wild-type virus nearly completely lacked the ability to infect human MSCs (Fig 4D).

**Fig 5:**
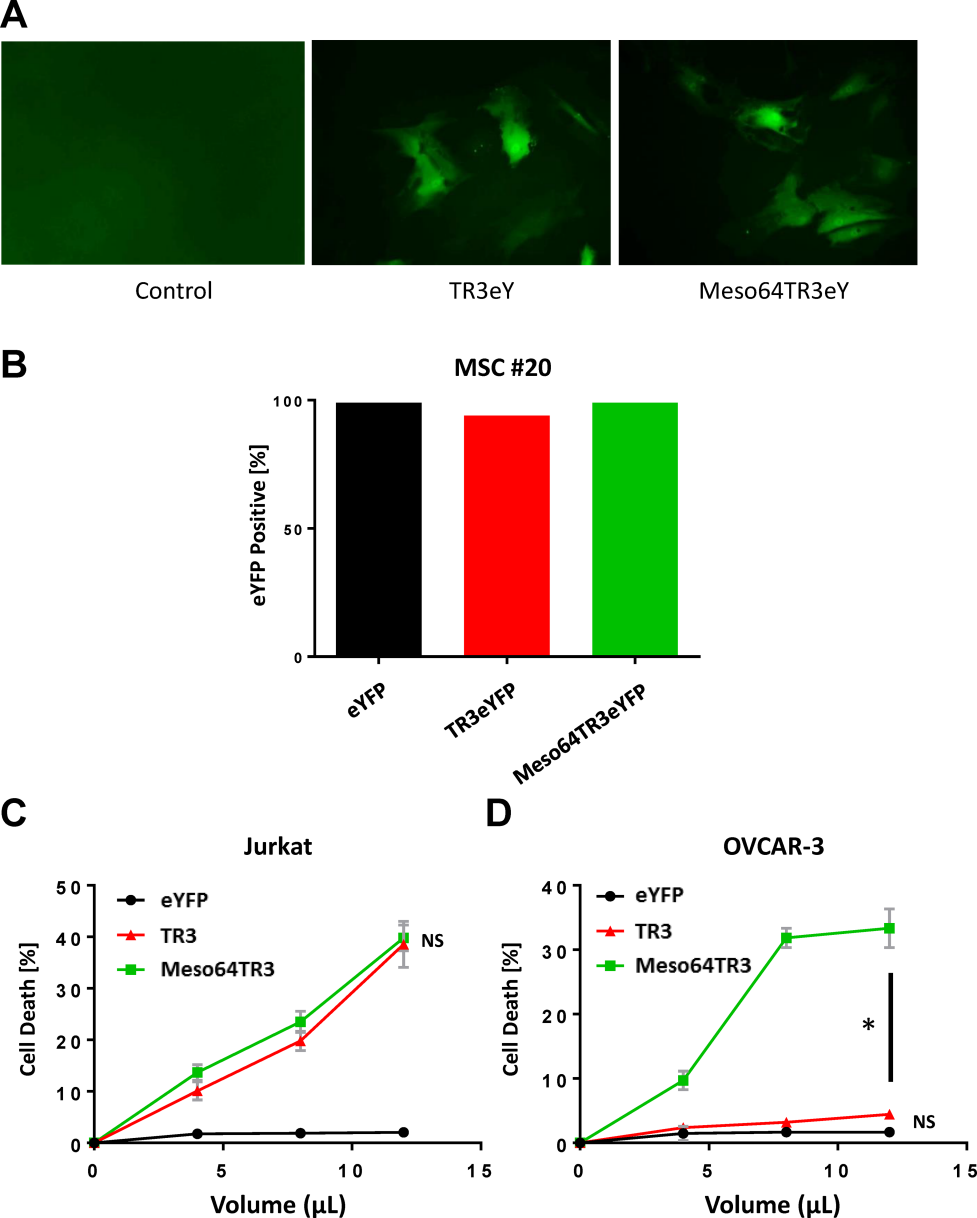
Ad5pK7 adenovirus can be “armed” with secreted forms of TR3-based cancer therapeutics. **(A)** Adipose-derived MSCs from patient #15 were infected with Ad5pK7 carrying TR3eYFP and Meso64TR3eYFP genomes using an MOI of 5000. Representative images were taken and show expression of eYFP fluorescence (original magnification 10x). **(B)** In order to produce secreted TR3 drugs for functional activity testing, plateau-reaching MOIs were used for the respective virus preparation (MSC #20, MOI 2500). These were subsequently confirmed by flow cytometry to ensure that infection rates resulted in equivalent production of the respective biologics. **(C)** Cell killing profiles of TR3 and Meso64TR3 at increasing drug volumes were established on MUC16-deficient T cell leukemia cell line Jurkat. Supernatant of cells infected with Ad5-eYFP was used as a control. Please note that both drugs induced a dose-dependent cell death overlapping response curves, consistent with their known activity profiles. NS, not significant. **(D)** The same cell death determination as in (C) using identical drug volumes was performed in MUC16-positive OVCAR3 cells. Please note the much enhanced activity profile of the MUC16-targeted cancer drug Meso64TR3 relative to parental TR3. NS, not significant; *, P < 0.04.

In an attempt to assess the feasibility of using MSCs as drug delivery vehicles based on MUC16-targeted TR3 therapeutics, we generated both TR3 and Meso64TR3-containing Ad5pK7 vectors. A bicistronic vector design was again used as described above, in order to assess transduction efficacy (Fig 1B). We confirmed that TR3 and Meso64TR3-containing expression units were capable of transducing human MSCs (Fig 5A). To test our hypothesis that MSC-derived Meso64TR3 would have enhanced activity profile against MUC16-positive OVCAR3 cells relative to parental TR3 and Ad5 control, we infected a randomly chosen MSC line (#20) with Ad5pK7 virus transducing TR3, Meso64TR3 and a YFP control. Three days post-infection, transduction efficacies were determined by flow cytometry and found to be of equal potency for all three viruses (Fig 5B). Supernatants derived from the same transduction experiment were then used to assess the ability of the MSCs to secrete the respective drug variants using cell viability assays. It turned out that TR3 and Meso64TR3 exhibited similar death-inducing capacity on MUC16-deficient Jurkat cells (Fig 5C). More importantly, when the same supernatants were tested on MUC16-positive OVCAR3 ovarian cancer cells, the MUC16-targeted Meso64TR3 variant was most efficient, while TR3 had almost no appreciable bioactivity, similar to the YFP control (Fig 5D). These data are remarkable in several ways. They are not only consistent with data we generated from CHO-derived supernatants above (see Fig 3), but they also reflect the differential drug characteristics between targeted and non-targeted TR3 in MUC16-expressing malignancies [17]. Encouraged by these *in vitro* results, we performed a small animal experiment and injected mice bearing subcutaneous OVCAR3 flank tumors (~100 mm^3^, n = 2) intraperitoneally with a single dose of YFP (control), TR3 and Meso64TR3-transduced MSCs (1 x 10^6^/mouse, ~100% transduction efficacies). Twenty-eight days post-MSC injection, we noticed a trend similar to what we had seen *in vitro*, in which tumors in the non-treated control grew the largest (1408 ± 600 mm^3^), followed by the TR3 group (357 ± 76 mm^3^) and Meso64TR3 (69 ± 25 mm^3^) (L. Kuroki and D. Spitzer, personal communication). These results were encouraging enough that were are currently in the process of designing a fully-controlled mouse experiment for the treatment of ovarian cancer with genetically engineered adipose-derived human MSCs secreting TR3-based therapeutics.

## Discussion

As our armamentarium of cancer therapeutics grows beyond standard cytoreductive surgery, chemotherapy, and radiation therapy, attractive therapeutic candidates that seem most promising include targeted approaches or strategic sequencing of drug administration that achieve synthetic lethality in cancer cells. A review by McLornan [44] highlights that synthetically lethal therapeutic approaches can exploit inherent differences between cancer and normal cells that is often not feasible with conventional chemotherapy. In line with these goals, our study highlights a unique approach to Ad-based drug delivery and cancer-specific cell death induction using autologous drug factories (MSCs) armed with targeted TR3-based cancer therapeutics; a concept highly dependent on efficient producer cell manipulation.

Due to the variable expression of the adenoviral CAR in human MSCs and primary ovarian cancer cells, the role of Ad vectors to enhance therapeutic approaches, including oncolytic viral therapy, has been constrained by the relative resistance to infection using Ad5-based vectors [23–27]. Prior strategies to bypass CAR deficiency have used integrin binding motifs and genetic capsid modifications. Specifically, the arginine-glycine-aspartate (RGD) and polylysine (pK7) motif have been shown to enhance Ad5 infection through an Ad5 receptor-independent pathway [36, 37]. Contreras et al [36] studied a double genetic modification with RGD/polylysine motifs and showed a significant reduction in the viral dose required to infect greater than 80% of pancreatic islet cells, resulting in reduced toxicity, inflammation and immune response. Another strategy studied by Kanerva et al [30] was to use an Ad5/3 chimera, which redirected binding of the vector to the Ad3 receptor. Exploiting the different tropism of Ad3 led to enhanced infectivity of MSCs, displaying a 5 to 16.5-fold higher transgene expression of Ad5/3 compared to Ad5 using four different primary patient-derived ovarian cancer cell lines.

In our current study, we demonstrated efficient transduction of MSCs with TR3-based therapeutics using a genetically modified Ad5 vector, Ad5pK7. Among previously published studies that evaluated MSC drug delivery utilizing conventional wild-type TRAIL or trimerizing monomer-based formats, the majority have either utilized an Ad [45–48] or a lentivirus [21, 22, 49–52] vector. However, among those that utilized an Ad vector, only one group [53, 54] reported transduction rates of 80% employing fiber knob modifications via incorporation of branched oligomeric cell-permeable peptides (CPPs) to achieve higher MSC infection rates. Regardless of vector type or commercial transfection kit employed, once the low rate of MSC transfection/transduction was overcome, studies have shown that TRAIL-expressing MSCs demonstrate impressive anti-tumor activities against mesothelioma [50], gliomas [51, 54–56], breast [52], renal [57], colon [48], and pancreatic [46] carcinoma xenografts, as well as inhibition of metastases [21, 45].

Beyond safety and feasibility of using MSCs as cellular carriers, application and effectiveness of TR3-armed MSCs in an ovarian cancer model are important aspects to address. For example, further investigating differences and advantages of secreted versus membrane-anchored TR3 constructs will help boost cell-specific killing and has potential to reduce off-target side effects. Interestingly, Moniri et al evaluated TRAIL-engineered pancreas-derived MSCs and showed that MSCs transfected with a secreted form of TRAIL showed more potent cell death than MSCs transfected with a non-secreted variant [46]. Such findings are the basis of our future studies to test Ad5pK7 transduction efficiency of MSCs using both membrane-anchored and secreted forms of TR3, where we will test this hypothesis using different cell lines *in vitro* and ultimately transition to preclinical mouse models of ovarian cancer.

It is equally important to address the fundamental principle that TRAIL-based therapy is highly dependent on the death receptor expression profiles of the tumor cells. So far, the majority of studies on MSC-mediated cytotherapy using TRAIL have been performed on pancreatic carcinoma [48]. However, we strongly believe that this model of drug delivery can also be applied to ovarian cancer due to our prior success in developing soluble, mesothelin-TR3 fusion proteins (MesoTR3 and Meso64TR3), which bind with high affinity to MUC16, highly expressed on ovarian cancer cells. In fact, we recently showed that this high-affinity ligand/receptor interaction was associated with a rapid and selective accumulation of MesoTR3 and Meso64TR3 on MUC16-expressing cancer targets. This directly correlated with increased killing activities *in vitro* and in xenograft mouse models of ovarian cancer and dominated over the TR3/death receptor interaction of the dual-domain therapeutic [16, 17]. In our current paper, we were able to achieve high transduction efficiencies of MSCs with TR3 and Meso64TR3, which we believe to be a key prerequisite for enhancing tumor-specific target cell elimination with genetically engineered drug factories *in situ*. In fact, when we tested the supernatants from MSCs infected with TR3 and Meso64TR3 *in vitro*, we could indeed confirm the ability of the MSCs to secrete functionally active biomolecules. More importantly, the characteristic activity profiles of both cancer drugs were retained in MUC16-deficient as well as in MUC16-positive ovarian cancer cells. Furthermore, preliminary data suggest that the MSC-derived cancer drugs will be efficient *in vivo* as well, even in a very stringent model system in which the tumor cells were established on the flanks of the mice, while the MSCs were injected into the abdomen of the animals.

As we continue to improve our technologies for the efficient transduction of MSCs and expand their applications as cellular carriers for targeted TR3 therapies, future directions will not only encompass combination with standard-of-care chemotherapeutics, but also focus on identifying patient populations who would benefit most from this innovative drug delivery system. In particular, studying factors such as histologic type, death receptor status, biomarker status (e.g. MUC16 and mesothelin), specific tumor genotypes, early versus advanced stage disease, and upfront versus recurrent setting, we may better elucidate the role and impact TR3 might have as a therapeutic in ovarian cancer. Furthermore, the optimal route, timing, and number of doses need to be further elucidated, as we can imagine a diverse number of methodologies to test TR3-expressing MSCs in ovarian cancer models. Equally important, techniques for evaluating cellular therapy also need to advance in parallel, which include multimodal imaging systems to assess cellular migration, proliferation, and overall function.

## Supporting information

S1 Fig Membrane localization of TR3GPI and TR3DAF. **(A)** CHO-CAR cells were either untreated (control) or infected with the membrane-anchored TR3 variants Ad5-TR3GPIeYFP (MOI 5000) and Ad5-TR3DAFeYFP (8750). Transduction efficacy was monitored via fluorescent protein expression (eYFP) and via anti-TRAIL surface staining (a-TRAIL/PE). Secondary antibody alone was used as a control (a-ms/PE). **(B)** Representative images of CHO-CAR cells 7 hours post-infection document the eYFP expression pattern via epifluorescence microscopy.
(TIF)
