# Supplementary figures and images for: Adenovirus platform enhances transduction efficiency of human mesenchymal stem cells: An opportunity for cellular carriers of targeted TRAIL-based TR3 biologics in ovarian cancer
Source: PLoS One. 2017 Dec 21;12(12):e0190125. doi: 10.1371/journal.pone.0190125 (PMC5739501; doi:10.1371/journal.pone.0190125)

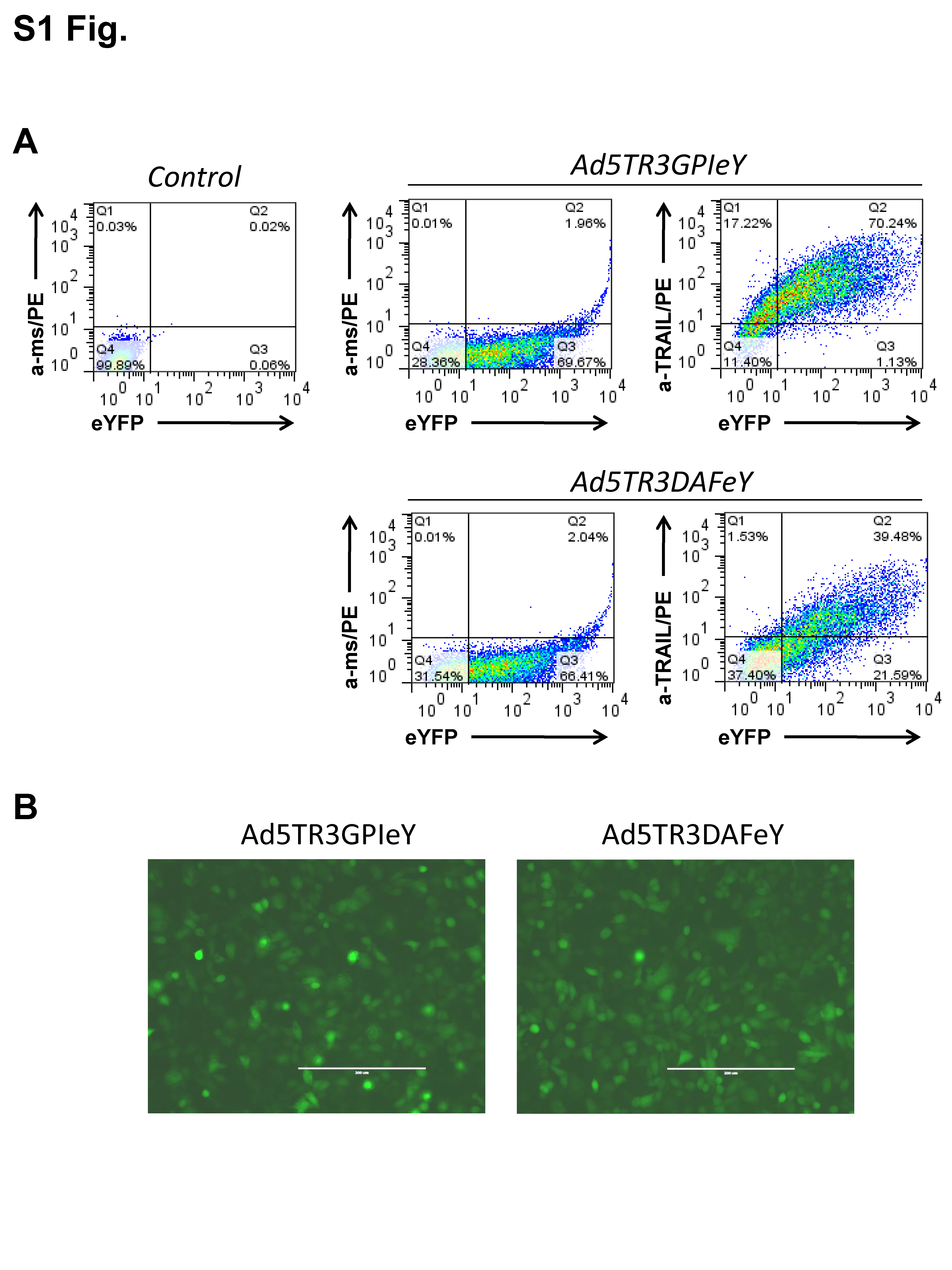

Supplement: S1 Fig — (A) CHO-CAR cells were either untreated (control) or infected with the membrane-anchored TR3 variants Ad5-TR3GPIeYFP (MOI 5000) and Ad5-TR3DAFeYFP (8750). Transduction efficacy was monitored via fluorescent protein expression (eYFP) and via anti-TRAIL surface staining (a-TRAIL/PE). Secondary antibody alone was used as a control (a-ms/PE). (B) Representative images of CHO-CAR cells 7 hours post-infection document the eYFP expression pattern via epifluorescence microscopy. (TIF) [file pone.0190125.s001.tif]
